# Microbial Colony Image Enhancement Algorithm Based on Composite Adversarial D-DCGAN Neural Network

**DOI:** 10.3390/microorganisms14092112

**Published:** 2026-09-21

**Authors:** Wangxin Li, Mingfeng Ge, Jing Sun, Dongxin Shi, Chenyu Jiang

**Affiliations:** 1 Division of Life Sciences and Medicine, School of Biomedical Engineering (Suzhou), University of Science and Technology of China, Hefei 230022, China; liwx@sibet.ac.cn; 2 Suzhou Institute of Biomedical Engineering and Technology, Chinese Academy of Sciences, Suzhou 215163, China; gemf@sibet.ac.cn (M.G.); sunj@sibet.ac.cn (J.S.); shidx@sibet.ac.cn (D.S.); 3 Shandong Key Laboratory of Digital Service Computing Technology and Systems, China, Jinan Guoke Medical Technology Development Co., Ltd., Jinan 250013, China

**Keywords:** microorganisms, compound DCGAN, recurrent neural network, dataset

## Abstract

Microbial testing plays a crucial role in medical and biological research, serving as a significant direction in disease prevention and control studies. Traditional manual testing, which involves identifying colonies on Petri dishes is the best method, but it is not only expensive but also time consuming. However, the application and development of deep learning can address these issues. However, a crucial condition is that deep learning models require a large amount of labeled medical imaging data. Obtaining high-resolution images is time consuming and cannot generate a large amount of data in a short time. Therefore, we designed a Double Deep Convolutional Generative Adversarial Networks (D-DCGAN) to construct a dataset. Under the DCGAN adversarial network, synthetic colonies are generated by overlapping small single colonies and composite colonies to establish a random synthetic colony model, and a DCGANB generation model is established for the synthesis of complete colonies. In this study, we first evaluated the D-DCGAN model, obtaining an initial FID score of 38.9, and validated the effectiveness of the generated dataset using Faster R-CNN and YOLOv12. Subsequently, a second-order optimization model based on multiple evaluation metrics was developed to determine the optimal augmentation level, identifying 1320 synthetic images from the original 236 samples. With this optimized dataset, the FID score decreased to 37.1, while the detection accuracy reached 0.975. These results demonstrate the feasibility of the proposed framework for effective dataset augmentation and improved downstream detection performance.

## 1. Introduction

Microbial culture and testing play important roles in medical diagnostics and biological research. Microorganisms of interest are typically cultivated on specific media, followed by microscopic observation and other detection techniques to identify and quantify microbial colonies. Microbial colony analysis is widely applied in clinical diagnosis, environmental monitoring, and food safety assessment [1,2]. In this study, we focus on culture-plate images acquired during microbial testing. *Escherichia coli* is an important model organism widely used in genetics and molecular biology. Pathogenic strains of *E. coli* are commonly transmitted through contaminated food or water and may cause severe intestinal or systemic infections. Notably, neonatal meningitis caused by *E. coli* (NMEC) accounts for approximately 28–29% of neonatal bacterial meningitis cases and is associated with a high risk of mortality [3]. Moreover, the isolation of *E. coli* from blood cultures has important clinical significance and is associated with severe infectious conditions, including sepsis and organ dysfunction [4,5]. Therefore, rapid and accurate detection of clinically important microorganisms such as *E. coli* is of considerable significance for clinical diagnosis and microbiological research.

Currently, the investigation and identification of *E. coli* in hospitals and research institutions generally rely on cultivation using biological sample media [6]. Conventional culture-based testing workflows are predominantly performed manually, requiring trained personnel to identify and count bacterial colonies after incubation [7]. This process is time consuming and labor intensive and may introduce counting errors due to operator fatigue. Consequently, image-based automated microbial colony recognition has emerged as an important alternative to conventional manual analysis [8]. The quantity and diversity of training images play a critical role in recognition performance [9]. Limited datasets can increase the risk of overfitting and reduce model generalization [10], whereas acquiring large numbers of high-quality microbial images through manual experimentation is costly and time consuming. This data scarcity represents a major challenge for automated microbial image recognition.

In microbial image recognition, the demand for large annotated datasets is particularly challenging because image acquisition and annotation require substantial experimental and labor costs. Deep learning-based visual recognition has achieved significant advances in computer vision [11,12,13,14,15], but its performance largely depends on sufficiently large and representative training datasets. Consequently, increasing the availability of high-quality training samples remains an important bottleneck for model optimization and practical applications [16].

To address limited-sample learning, conventional data augmentation methods, including rotation, color transformation, cropping, and jittering, have been widely used [17,18,19]. However, these operations mainly generate transformed versions of existing images and therefore retain strong correlations with the original samples, limiting their ability to expand higher-order features such as complex colony morphology, density, and spatial distribution. Other image-enhancement techniques, including sharpening, histogram equalization, and white balancing, similarly rely on spatial- or pixel-level transformations [20,21,22]. Although synthetic image generation provides an alternative approach to dataset augmentation [23], existing methods may still have limited capability in representing the high-level structural diversity required for complex microbial colony images.

Generative Adversarial Networks (GANs) learn the underlying data distribution through adversarial training between a generator and a discriminator and have demonstrated considerable potential in medical image synthesis, biomedical image augmentation, and pathogen-related image analysis [24,25]. However, conventional GANs remain susceptible to mode collapse, training instability, and convergence difficulties, which can affect the diversity and realism of generated images. Therefore, an effective augmentation framework for microbial colony images should simultaneously improve image realism and diversity while ensuring the usefulness of synthetic data for downstream recognition tasks [26].

To address these challenges, we propose a Random Composite Microbial Data Augmentation Generative Adversarial Network (D-DCGAN). The proposed framework employs a composite DCGAN architecture to generate high-quality multi-colony microbial images from limited training samples in an unsupervised manner without requiring paired image data. By learning the underlying distribution of microbial colony images, D-DCGAN generates synthetic samples with diverse colony characteristics and spatial configurations. To further improve generation quality and determine the optimal augmentation level, we introduce a D-DCGAN evaluation and optimization module based on a second-order regression model. The effectiveness of the augmented dataset is further validated using Faster R-CNN and YOLOv12. Experimental results demonstrate that D-DCGAN can effectively expand limited microbial colony image datasets and improve the detection performance of downstream microbial recognition models, providing an effective data augmentation strategy for intelligent microbial image analysis under small-sample conditions.

## 2. Data and Methods

In this section, we provide an overview of the production process of image data and classic data augmentation methods.

### 2.1. Escherichia coli Colony Culture

To prepare a small-scale dataset of *Escherichia coli* culture-plate images for this study, *E. coli* ATCC 25922 was used as the experimental strain [27]. Luria–Bertani (LB) medium was used for bacterial cultivation. Solid culture medium was prepared by dissolving LB powder and agar in distilled water at concentrations of 25 g/L and 15 g/L, respectively. The mixture was thoroughly homogenized using a magnetic stirrer (DragonLab, Shenzhen, China) and subsequently sterilized in an autoclave at 121 °C for 15 min. After sterilization, the medium was allowed to cool to approximately 50 °C, after which an appropriate amount of antibiotic was added when required. The sterilized medium was then poured aseptically into sterile Petri dishes at a volume of approximately 15–20 mL per plate, corresponding to approximately one-third of the dish depth. The prepared plates were stored at 4 °C until use. For bacterial isolation, a small amount of *E. coli* culture or an individual colony was collected using a sterile inoculating loop and streaked onto the surface of the prepared solid medium using the standard plate-streaking method to facilitate the isolation of individual colonies. The plates were subsequently incubated in an inverted position at 37 °C for 12–16 h. After incubation, the formation and morphology of individual colonies were examined. The resulting solid cultures were used for colony observation, isolation, and image acquisition. To obtain culture plates with different bacterial concentrations, bacterial suspensions at dilution levels of 10^−6^ and 10^−7^ were prepared and used for culture-plate preparation. A total of 236 Petri dishes were prepared and incubated under the corresponding experimental conditions. Images of the resulting microbial colonies were acquired at 24–48 h after incubation and subsequently used to construct the microbial colony image dataset for the subsequent image-generation and data-augmentation experiments.

### 2.2. Bacterial Image Acquisition and Preprocessing

**Figure 1:**
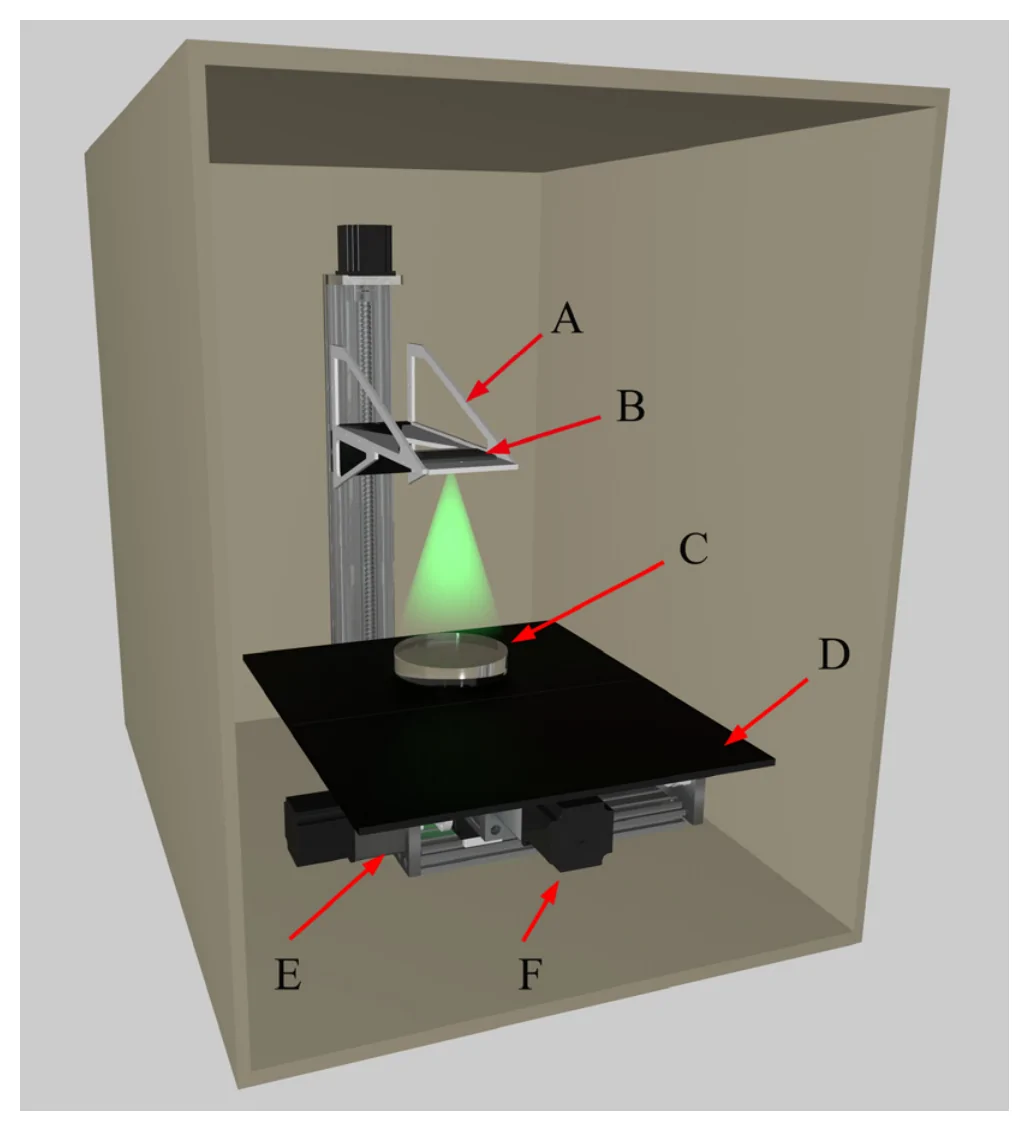
Schematic diagram of colony image equipment. (A) Fixed support. (B) Imaging mobile device. (C) Culture medium. (D) Sample platform. (E) *X*-axis motor. (F) *Y*-axis motor.

An integrated lighting system was employed to capture images of *Escherichia coli* colonies, which involved utilizing a mobile imaging device (Honor90, Honor, Shenzhen, China) and a stepper motor-driven mobile platform capable of moving along both the *X*-axis and *Y*-axis. One LED backlight source and a closed shading box are used to isolate and interfere with external light. The built-in mobile phone collection and lighting equipment have a pixel resolution of 4032 × 3024 isotropic pixels (0.029 mm/pixel point). The specific structure is shown in Figure 1. To obtain clear and distortion-free images, A fixed imaging device with a *Y*-axis length of 280 mm was installed. The camera is fixed on the platform and was ensured to be parallel to the Petri dish, with an appropriate focal length. The focal length for height was adjusted and calibrated. Finally, the overall height of the device was adjusted to 405 mm and image data acquisition was continued. Under the influence of reflection from the point light source and the turning of the culture medium cover, the collected images of *Escherichia coli* were corrected. The light generated by the image underwent white balance calibration, grayscale world algorithm calibration, and simple image calibration. The image channels C(R,G,B) were calibrated according to Formula (1), with the G channel as the reference, to obtain the calibrated image Cnew. During the cultivation process of 236 Petri dishes, 236 colony images were collected. Furthermore, the performance of D-DCGAN was evaluated.

(1) $$C_{\mathrm{new}}\left(x,y\right)=C\left(x,y\right)\times \frac{\mathrm{mean}\left(G\right)}{\mathrm{mean}\left(C\right)}$$

### 2.3. Introduction to Adversarial Network Algorithms

#### 2.3.1. Generative Adversarial Network Algorithm GAN

**Figure 2:**
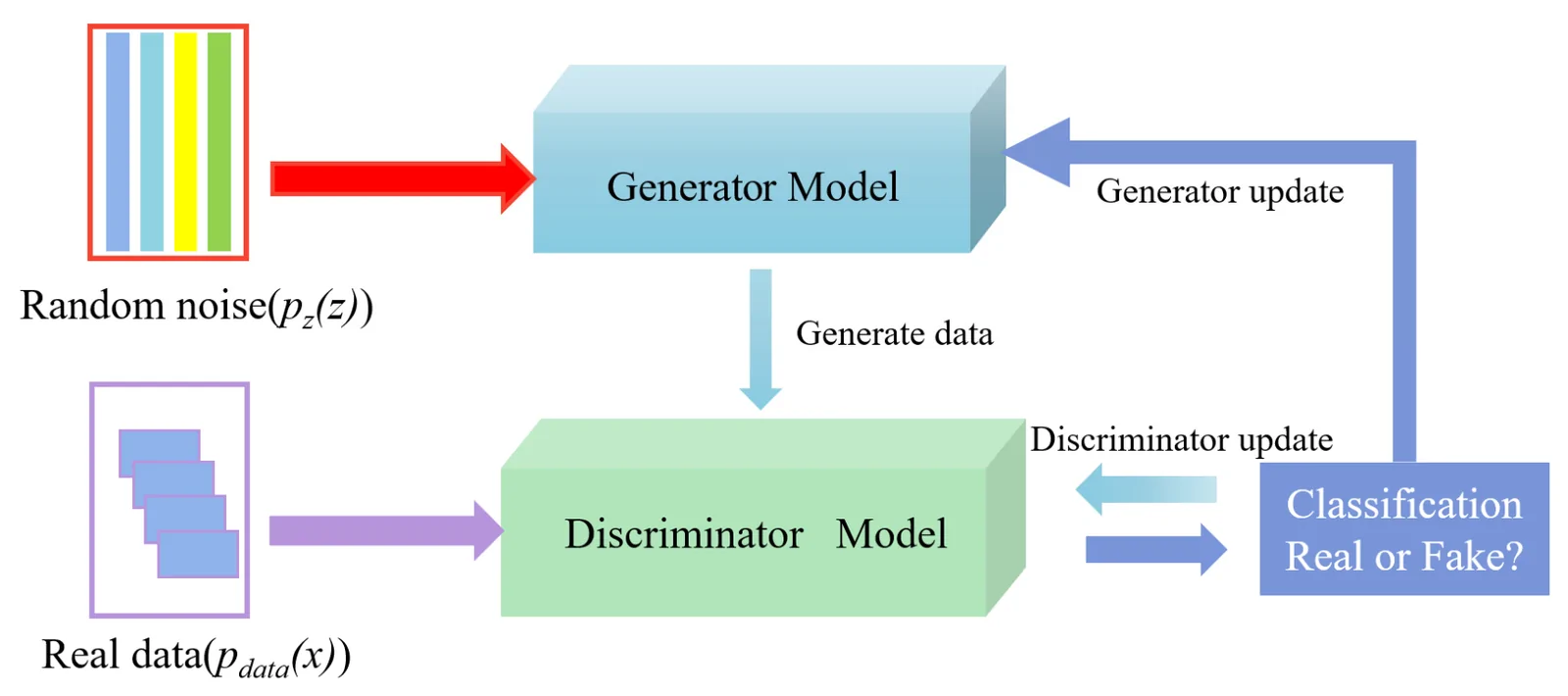
The structure and working principle of the Generative Adversarial Network (GAN). The GAN consists of two main components: a generator and a discriminator, which are trained through an adversarial learning process.

In the early days, Generative Adversarial Networks (GANs) were a type of deep learning algorithm for image enhancement. The structure is shown in Figure 2. In 2014, Ian Goodfellow proposed an algorithm for augmenting and enhancing datasets [28], which primarily involves adversarial computation based on two independently calculated networks: the generator (G) and the discriminator (D). The training of the generator (G) and the discriminator (D) involves a zero-sum game. The goal of the generator is to confuse the discriminator by producing increasingly realistic fake data, while the discriminator aims to enhance its discriminative ability by identifying the fake data. Through this competitive process, the generator is ultimately able to generate highly realistic data. The generator network and the adversarial network engage in an adversarial process, ultimately reaching a further equilibrium state (Nash equilibrium).

(2) minGmaxDVD,G=Ex∼pdata xlogDx+Ez∼pzzlog1−DGz

*x* represents the real image, *p_data_*(*x*) represents the distribution of real data from which a real image is drawn, *z* is the input noise in the generator G, and *p_z_*(*z*) is the distribution of the noise. The GAN network generates the G distribution based on distribution and obtains noise distribution through z. In the process of distinguishing generated samples from real samples in the loss function (2) of the discriminator D, the generator network takes random noise as its initial state and is then discriminated by the discriminator. The binary cross-entropy loss function is calculated to further optimize the generator and loss function and update and calculate the discriminator gradient. Under the premise of traditional GAN technology application, gradient vanishing or explosion may occur. In simple GAN network training methods, gradient descent is performed on the gradient parameters [29,30].

#### 2.3.2. Deep Convolutional Generative Adversarial Network (DCGAN)

**Figure 3:**
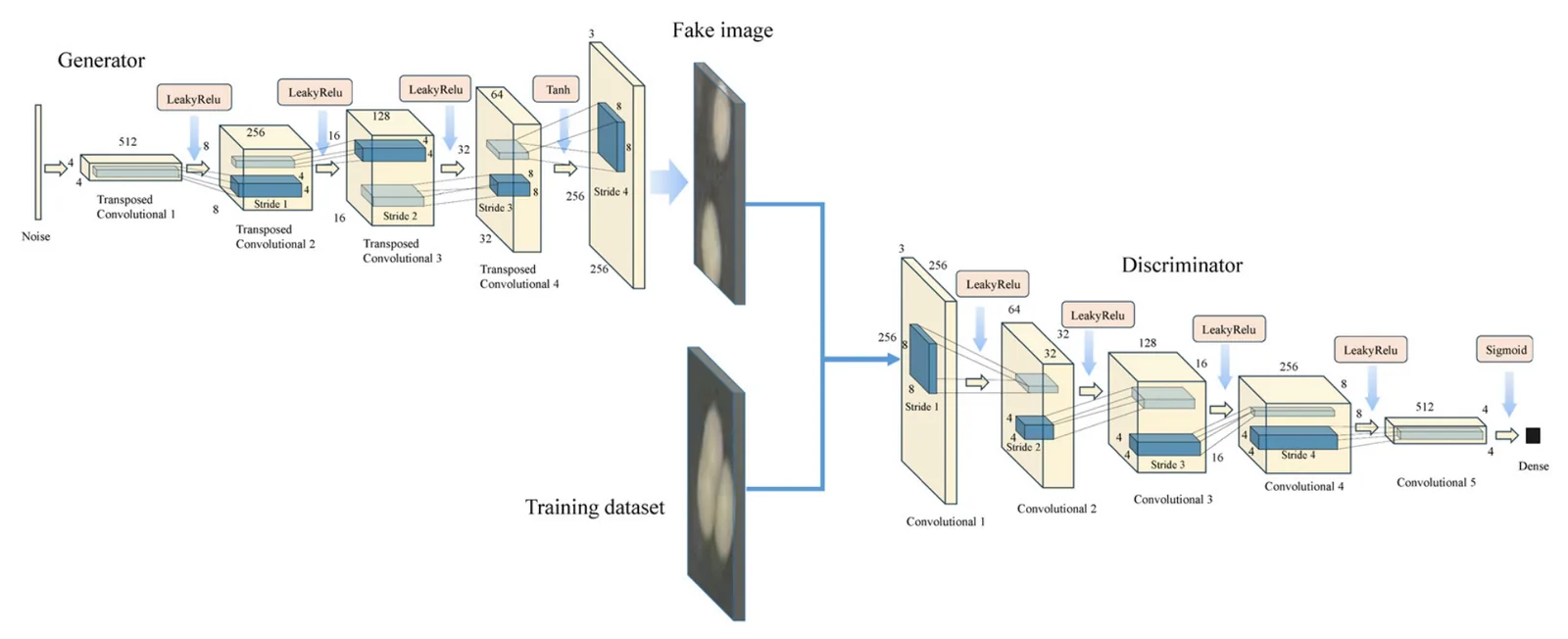
Schematic architecture of the deep convolutional generative adversarial network (DCGAN).

The DCGAN (Deep Convolutional Generative Adversarial Network) is a variant form of the GAN (Generative Adversarial Network), as shown in Figure 3. By introducing the Convolutional Neural Network (CNN) structure, the performance of both the generator and the discriminator is enhanced, exhibiting significant advantages in handling high-dimensional data. This approach replaces traditional fully connected network structures with convolutional upsampling and deconvolutional downsampling [31,32]. In DCGAN, unsupervised learning is performed on D and G through convolutional layers. In the computation of small datasets, the discriminator expands the data (256 × 256 × 3), computes the model using the LeakyReLU activation function to avoid the Dead ReLU phenomenon. Batch normalization is applied to the image data to accelerate training and stabilize the model. The Sigmoid activation function is used to map the output to the range of 0 to 1, indicating the authenticity of the input image. The generator performs transposed convolution processing on the input noise signal to generate data signals. In this architecture, the batch normalization layer is used to perform batch normalization after single-layer computation with a Sigmoid activation output function.

#### 2.3.3. Improved Composite Adversarial Network D-DCGAN

**Figure 4:**
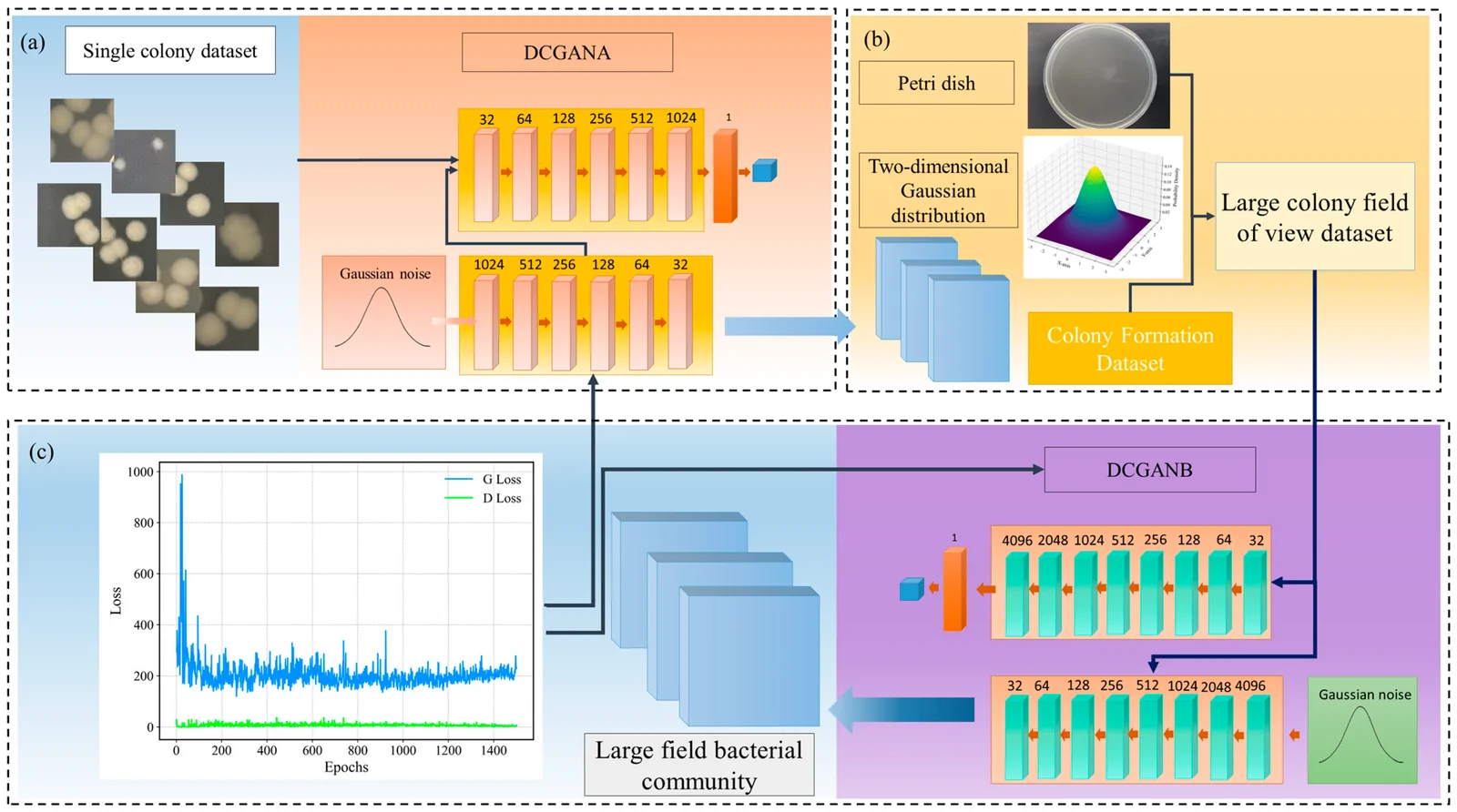
Computational architecture and workflow of the Composite D-DCGAN model. (**a**) Single colony generation via DCGANA, (**b**) spatial distribution modeling, (**c**) large-field bacterial community synthesis and network training.

In the computation process of deep learning algorithms, when dealing with large images with high resolution and a large number of pixels, there are significant difficulties in generating images with high resolution. During the generation process of *Escherichia coli*, high-quality bacterial images are gradually generated through a hierarchical DCGAN, and the generated bacterial images are then synthesized. The DCGANA generates data for single colonies, based on the original small graphics of single colonies, by segmenting and processing the high-resolution large images from the original dataset. When the sample size is small, it generates data for single colonies and conducts evaluation tests on the data. In the case of a small and incomplete original dataset, an excessively small number of samples can easily lead to overfitting, and a lack of features can result in poor image quality, ultimately causing the generated images to be distorted [33]. A small dataset is generated based on the existing image segmentation foundation, and preliminary data augmentation is performed using DCGAN to expand the dataset. The overall structure is shown in Figure 4.

In DCGANA, the emphasis is primarily placed on the processing of small-sized samples. The input source is small colony images, which undergo data segmentation. Single-bacteria and multi-bacteria small images (3 × 256 × 256) are inputted along with random noise z to construct an upsampling layer for deconvolution network. The Relu activation function is used for activation computation. The main network structure is a six-layer transposed convolutional network with a stride of 4, a kernel size of 4, and 1024 input channels. The data proceeds to the next step with a stride of 4 and a kernel of 4, and further repeats to complete the data computation of the generator. DCGANA is used to generate a small-sized high-resolution image set from random noise. Random noise is input into the generator to create small synthetic images. Based on the probability distribution, the variability is further determined, the data are optimized, and preliminary data augmentation is achieved.

In DCGANB, the generator is further adjusted during the augmentation of data volume. It generates a large-sized dataset from a small-sized dataset obtained by DCGANA, under the state of a random number set, and then proceeds with generation. The original data and synthetic data it utilizes generate random large-scale data of multiple colonies, with the generated synthetic multi-colony data serving as input. Pixels are converted based on the training set, and a discriminator is used to distinguish between real images and synthetic images. Generation computation is performed on the synthetic multi-colony image, with a size of (2048 × 2048 × 3). The generator computes noise (1 × 1 × 100). An enhanced deep DCGANB is employed for processing, and the data undergo transposed convolutional layer processing with weights. One of the transposed convolution layers is activated by the ReLU function and merged. The next step involves the simultaneous processing of dual transposed convolution layers, during which the output and channel are fixed. Finally, the generated data is formed through an activation function, resulting in a random big data bacterial colony dataset.

### 2.4. Quality Assessment of Composite Data

During DCGAN processing, the divergence distance between distributions is reduced [34]. To evaluate the divergence distances, we adopted method indicators from GAN algorithm data evaluation in this study to determine reliability, including Fréchet Inception Distance(FID) [35], mean structural similarity (SSIM) [36], and the Peak Signal-to-Noise Ratio (PSNR) evaluation method [37]. FID is primarily used to measure the feature distance between two sets of generated images and real images. In this research process, a composite algorithm combining DCGANA and DCGANB is adopted, utilizing the feature extraction of FID in a sequential manner. Features from it are extracted using the original weights of the Inception v3 network, and all images are resized to a uniform size. Features from real images and generated images are extracted using a pre-trained Inception v3 network, and feature vectors through the Inception network are obtained. These feature vectors are typically high-dimensional. In the FID evaluation calculation, the true feature mean and covariance of the bacterial images, as well as the feature mean and covariance of the generated images, are extracted to compute the FID distance (F). Here, Tr represents the trace of a matrix, which is the sum of the diagonal elements of the matrix. *μ_r_* and *Σ_r_* represent the feature mean and covariance of the real bacterial image, respectively, while *μ_g_*, *Σ_g_* represent the mean and covariance of the generated image, respectively. The smaller the FID value, the more similar the generated images are in the high-dimensional feature space, indicating better image quality.

(3) $$\mathrm{FID}={\left\|\mu_{r}-\mu_{g}\right\|}^{2}+\mathrm{Tr}\left(\Sigma_{r}+\Sigma_{g}-2{\left(\Sigma_{r}\Sigma_{g}\right)}^{1/2}\right)$$

The SSIM (Structural Similarity Index) is a metric used to measure the visual similarity between two images, aiming to better simulate how the human visual system evaluates image quality. A structural assessment of data quality is conducted, and the adaptability of bacterial image structural features and their conformity to human visual perception are evaluated. The similarities of three characteristics of bacterial images are compared: brightness, contrast, and structure, where x and y represent the pixel values of the image, respectively. *μ_x_* and *μ_y_* represent the means of images x and y, respectively, *σ_x_* and *σ_y_* denote the variances of images x and y, respectively, *σ_xy_* signifies the covariance, *C*_1_ and *C*_2_ are constants, and L stands for the dynamic range of the image. With K_1_ = 0.01 and K_2_ = 0.03, the SSIM approaches 1, indicating that the two images are very similar in terms of structure, brightness, and contrast.

(4) $$\mathrm{SSIM}\left(x,y\right)=\frac{\left(2\mu_{x}\mu_{y}+C_{1}\right)\left(2\sigma_{\mathrm{xy}}+C_{2}\right)}{\left(\mu_{x}^{2}+\mu_{y}^{2}+C_{1}\right)\left(\sigma_{x}^{2}+\sigma_{y}^{2}+C_{2}\right)}$$

The Peak Signal-to-Noise Ratio (PSNR) is used to quantitatively evaluate the reconstruction quality between generated data and real data. PSNR measures image quality by comparing the pixel errors between the original image and the generated image, and its calculation is based on the Mean Squared Error (MSE). Assuming the original image is denoted as I and the generated image as K, with an image size of m×n, the mean squared error is calculated as follows:

(5) $$\mathrm{MSE}=\frac{1}{\mathrm{mn}}\sum_{i=1}^{m}\sum_{j=1}^{n}({I_{\mathrm{ij}}-K_{\mathrm{ij}})}^{2}$$

Further PSNR calculation:

(6) $$\mathrm{PSNR}=10\cdot \log(\frac{{\mathrm{MAX}}_{I}^{2}}{\mathrm{MSE}})$$

The evaluation models of three categories compare the similarity between the generated real data of DCGANA and DCGANB. They can adjust and optimize the overall performance of generated data for different DCGAN parameters, forming a cross-promoting DCGAN optimization.

## 3. Experimental Training

### 3.1. Test Environment

In the computation process, image processing is carried out, and data calculations are performed based on Pycharm 2024.2.1 and Python 3.9. Design and training of DCGANA and DCGANB are conducted. All software calculations are executed on a Win10 64-bit operating system, utilizing 32 GB of memory, an Intel(R) Core(TM) @i9-4770 CPU at 3.40 GHz, and an Nvidia GeForce RTX 4070 SUPER GPU with 12 GB of video memory.

### 3.2. Dataset

**Figure 5:**
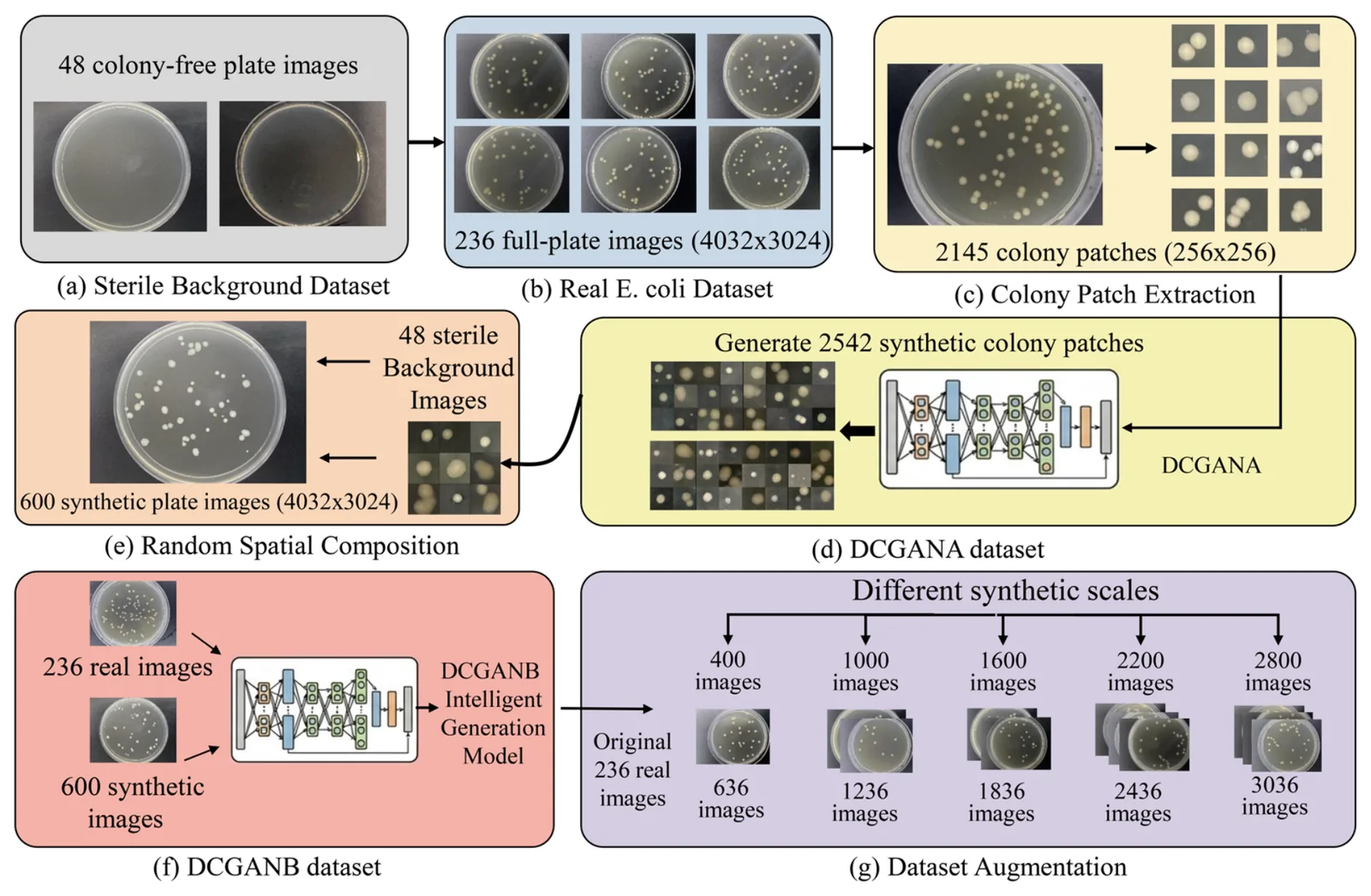
Dataset structure diagram. (**a**) Sterile background dataset. (**b**) Real *E. coli* dataset. (**c**) Colony patch extraction. (**e**) Random spatial composition. (**d**) DCGANA dataset. (**f**) DCGANB dataset. (**g**) Dataset augmentation.

In this research, we utilized images obtained from *Escherichia coli* colony samples cultured in the laboratory. *E. coli* underwent colony cultivation for 24–48 h at both high and low concentrations. The dataset structure is shown in Figure 5. Through an image acquisition device, we collected 48 images from the colony culture dishes and 236 images of the colonies. Additionally, we calibrated the 236 *E. coli* data points. We segmented the bacterial Petri dish image data from the on-site images to obtain 2145 single-bacteria and multi-bacteria images with a resolution of 256 × 256, and then performed data processing on them using DCGANA. We organized and combined images of small bacteria, as well as different datasets arranged for training the system. Based on a random 3:1 data split, the first group consisted of 1576 single-colony images, which were used for DCGAN to learn basic morphological features and generate diverse single colonies. Subsequently, DCGANB is relied upon to create multiple random colonies in a Petri dish background. For DCGANA training, the size of these images is reduced to 256 × 256. Different training datasets for DCGANA are generated, and through the training dataset of single colonies, a dataset of individual Petri dishes is formed relying on the synthesis method of random numbers. Furthermore, a multi-image dataset is formed through the data images of Petri dishes.

Initially, the data of single colonies was trained and processed. During the DCGANA cycle generation process of single colonies, the generator GA and discriminator DA were designed to train and generate all single colony images. Mini-batch training was adopted, with each batch of 32 images serving as the capacity of the mini-batch dataset for processing. The weights of GA and DA are continuously updated, and after multiple epochs of training, a better effect is achieved. During the training process of the discriminator DA, D(x) is calculated from the real dataset, while D(G(z)) is calculated from the generated image vector matrix, and the loss function is calculated to update the weights of the discriminator DA. The generated image G(z) produced by the generator is subjected to loss calculation by the discriminator, further ensuring that the generated image is close to the real image. The loss is calculated and the generator is updated.

To establish the optimal settings, the parameters of LearnRate, Batch Normalization, Leaky ReLU, and the Adam optimizer were determined based on the recommended parameters from the original DCGAN framework. We conducted extensive training and learning using DCGANA, and performed comparative studies on the learning progress of different DCGANA models. In the training setup, we calculated for 100, 500, 1000, 1500, and 2000 Epochs, and evaluated the gradual optimization of the generation of single colonies and multiple composite colonies. The loss function gradually decreased, and the losses of both the generator and the discriminator gradually decreased, indicating convergence of both. The data model of the DCGANA training structure is shown in Table 1.

**Figure 6:**
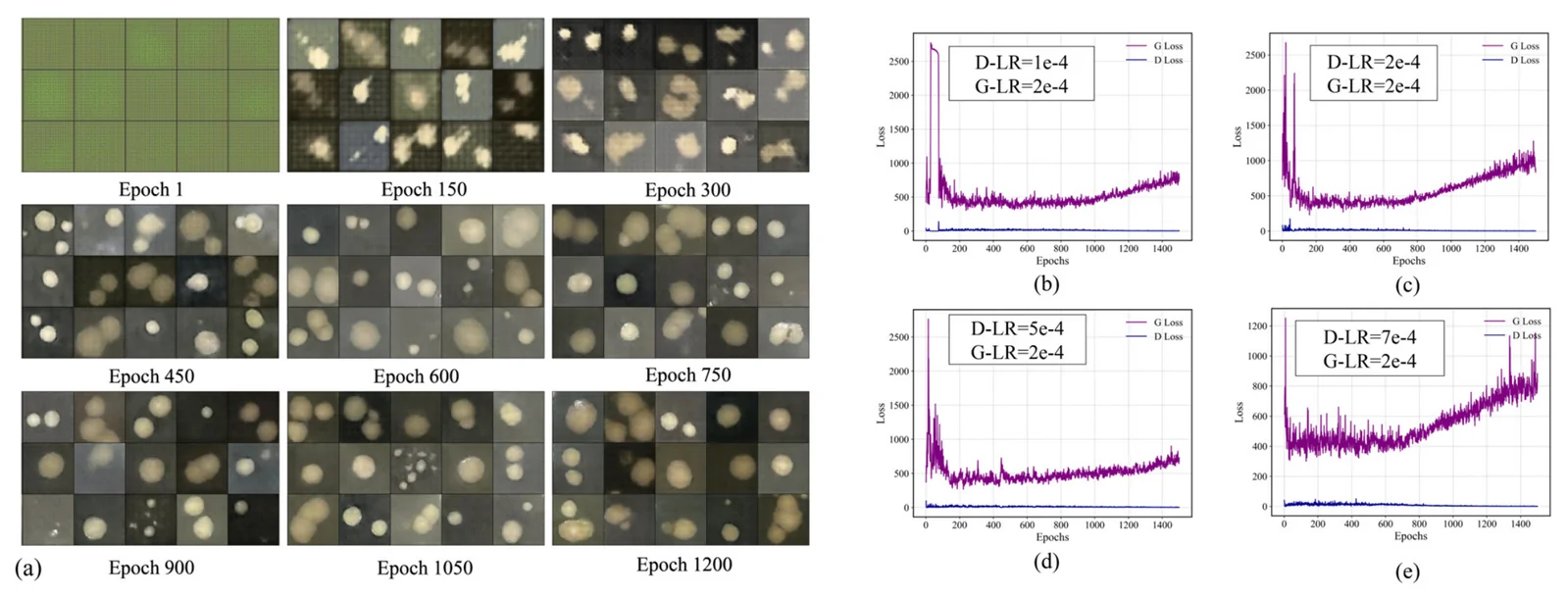
DCGANA computation process diagram. (**a**) Single colony training process. (**b**–**e**) Learning effects of different discriminators and generators with different learning rates.

The results obtained during the calculation process are shown in Figure 6. In this process, the colony formation effects under different Epoch designs are presented. To demonstrate the changing process of the generator and discriminator of DCGANA, Figure 6b–e show the variation of the loss values of the two components. Initially, there was obvious oscillatory variation, with significant changes in the loss value of the generator. As training progressed, the loss values gradually tended towards a stable state. We conducted tests with different learning rates, adjusting the learning rate of the discriminator while keeping that of the generator constant. It was found that when the learning rate of the discriminator was increased to 7 × 10^−4^, the loss value of the generator significantly increased and became unstable during training iterations of 800–1000. Therefore, during the training process, learning rates of 2 × 10^−4^ and 1 × 10^−4^ were used as the main training parameters for training experiments. The image effects generated during training are shown in Figure 6a, and the changes in computation rate under different learning rates of the generator and discriminator are shown in Figure 6b–e.

### 3.3. Random Simulation Data Generation

**Figure 7:**
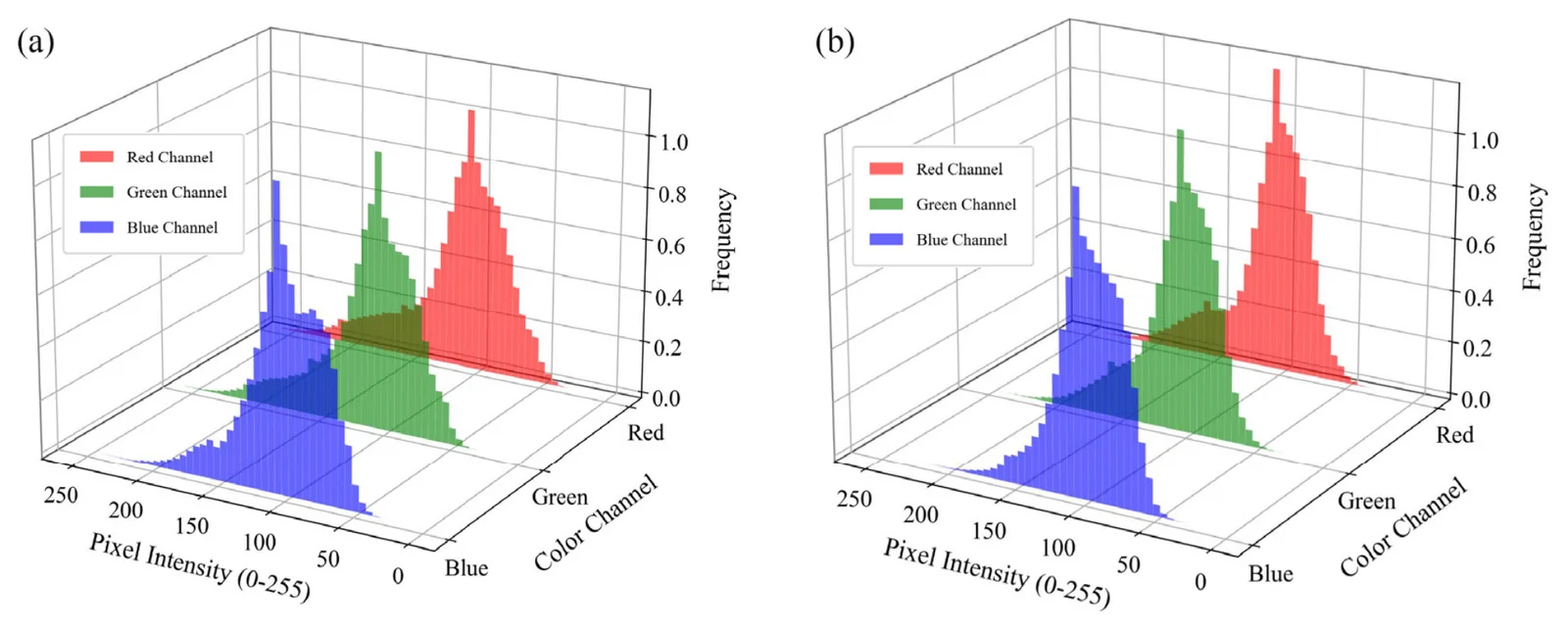
Pixel map of dataset statistics. (**a**) Pixel map of original dataset. (**b**) Pixel map of generated dataset.

In the small data image dataset generated by DCGANA, which includes single colonies and combined multiple colonies, the generated dataset was utilized to test its randomness and specificity. The dataset of single colonies underwent random specificity testing, and the generated plaque effect in the plaque images was tested for consistency in pixel distribution. An analysis was conducted on the 1750 images generated by DCGANA, and the statistical pixel maps are shown in Figure 7a,b. The pixel distribution structures are essentially identical, demonstrating consistency.

**Figure 8:**
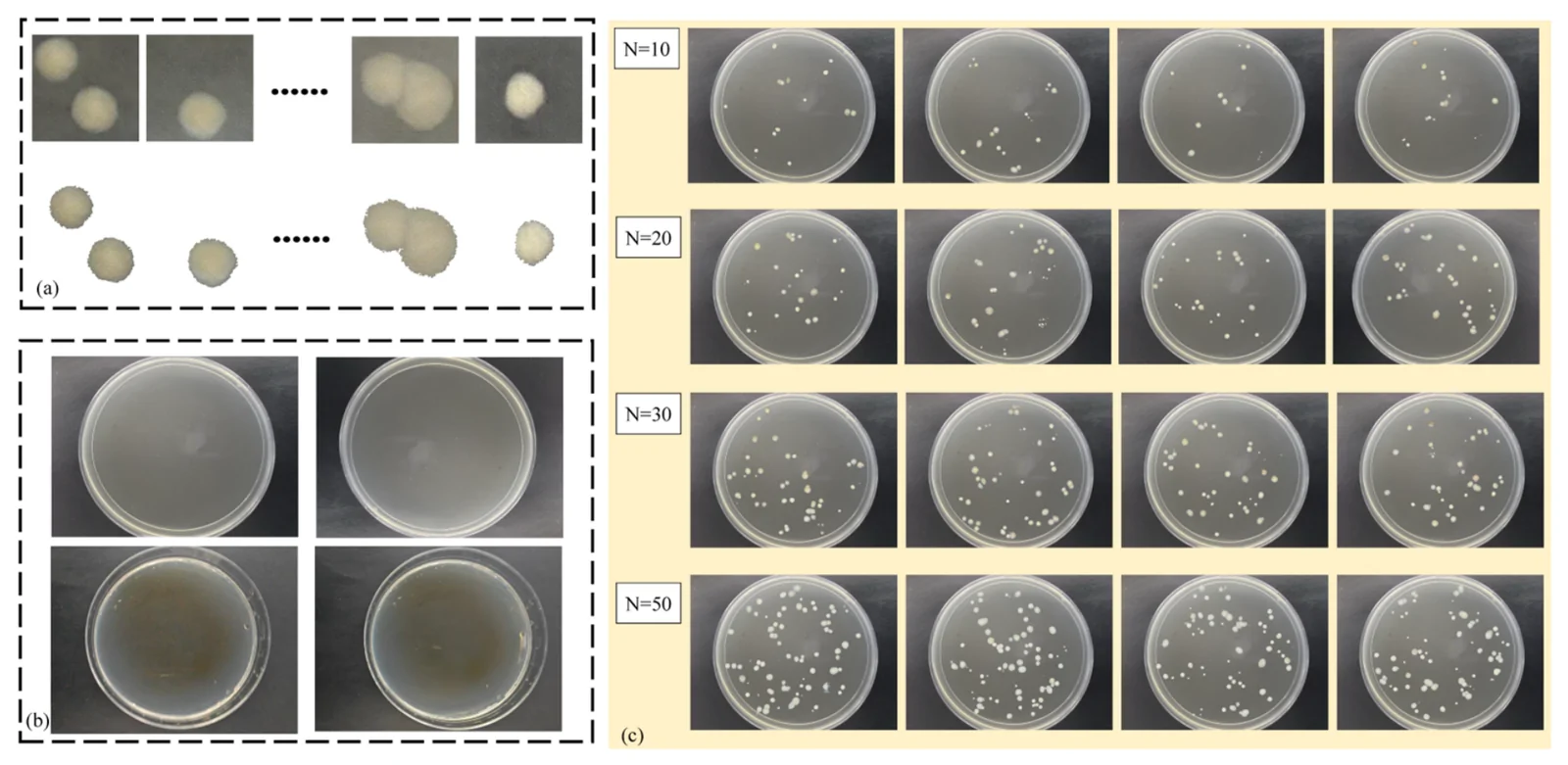
Generated effect diagram of random dataset. (**a**) Dataset of single bacterial colony. (**b**) Culture medium module diagram. (**c**) Generated random datasets with different quantities.

Under the condition of determining randomness, we relied on a random background template following a Gaussian random distribution. Initially, we employed the generative DCGANA model to remove the background of both single and multiple colonies, determining the coordinates through Gaussian distribution and randomly positioning them. We then extracted the colonies from the single-colony dataset using the center of the coordinates as the positioning base point for coordinate positioning, resulting in a mixed colony image that serves as the evaluation dataset. Based on this dataset, a multi-colony culture dataset is further formed. Randomness discrimination and similarity discrimination were conducted. Calculations are performed based on 10 samples, 20 samples, 30 samples, and 50 samples to determine the randomness of the samples. We initially generated single colonies, continuously and randomly generating colonies of bacteria in a Petri dish for *Escherichia coli*, simulating the final growth effect. For the generation experiment test on 10 sample data colonies, the image generation effect is shown in Figure 8. Figure 8a shows the generation of a single colony (within the range of [0:255]). In areas where the pixel color is similar to the average color of the patch background, the value of such a mask is lower. The idea of this step is to increase opacity (reduce the alpha channel) in areas that are similar to the background and contain less interesting information. This step can significantly enhance the visual realism of the generated patch.

In Figure 8b, we can see the image of a Petri dish generated by DCGANA through a random model. With limited data, we segmented and processed the original bacterial images to form a limited sample. Through the combination of culture media, we were able to generate random samples after growth. By further combining with DCGANB, at Epoch 1500, the D-Loss and G-Loss in the loss function were basically in a balanced convergence state, without overfitting, allowing for the combination and generation of random model images.

### 3.4. DCGANB Training

After completing the training process of DCGANA, we generated a similar dataset of single and dual bacterial colonies by cycling through DCGANA. Leveraging this dataset, we randomly combined multiple different colony images to form a diverse set of colony images, as shown in Figure 8c. By further combining the original limited overall dataset with the randomly combined dataset generated by the DCGANA model, a new dataset was formed. The DCGANB model was designed for adversarial training. Training was conducted in mini-batch mode, with each batch consisting of 16 images. The original 236 colony images and the 600 images jointly generated by DCGANA and the random model were used for training DCGANB, and the generated results were recorded and analyzed. The data model of the DCGANB training structure is shown in Table 2.

**Figure 9:**
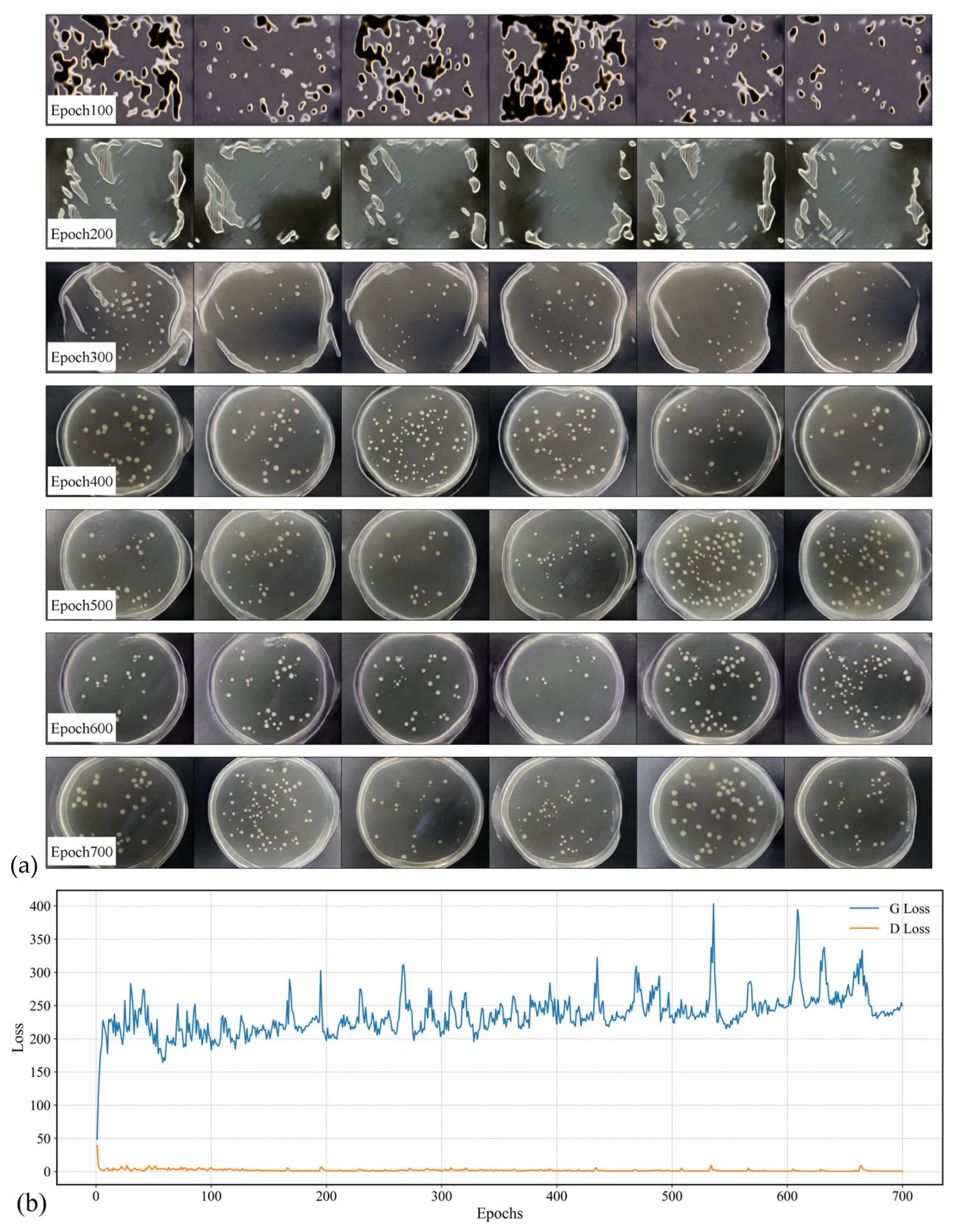
DCGANB training computation process. (**a**) Complete colony generation process. (**b**) Loss variation of generator and discriminator during complete colony generation.

In this experiment, the image generation process of DCGAN-B was systematically recorded, and the resulting images were quantitatively analyzed. Representative generated images are presented in Figure 9a. Comparative analysis demonstrated that incorporating CNN-based architectural components into the GAN framework substantially improved the efficiency and quality of image generation. The combined architecture exhibited a stronger ability to approximate the underlying distribution of real microbial colony images, while generating samples with greater morphological and spatial diversity. Overall, the results demonstrate that DCGAN-B can effectively generate diverse synthetic bacterial colony images and provide an efficient approach for expanding the microbial colony image dataset. The results show that during the DCGANB generation process of bacterial culture colonies, the colonies are generated clearly. From an observational perspective, the main colony structures are achieved, but there is a certain degree of irregularity in the Petri dishes. Further evaluation of the generated dataset of these colonies is needed to determine the generality of the dataset features. It was found that the shape rules generated at the edge contours did not achieve a smooth curvature, mainly due to the deficiency in the number of random datasets.

## 4. Results

### 4.1. Quantitative Evaluation

Considering the evaluation of generated data quality based on the loss function of the Generative Adversarial Network (GAN), an assessment of the accuracy of data generated by the DCGANB model needed further determination. This study employed a quantitative evaluation of the generated data. Through the joint analysis of DCGANA and DCGANB, the expansion of the bacterial colony dataset was achieved, and their generative characteristics and effects were analyzed. To quantitatively evaluate the effectiveness of DCGAN-generated data, this study utilized the Frechet Inception Distance (FID), Structural Similarity Index (SSIM), and Peak Signal-to-Noise Ratio (PSNR) indicators to assess the quality and diversity of the generated samples.

We evaluated and compared datasets of original colonies, randomly generated colonies, and colony generated by adversarial network DCGANB. The comparison of these three results is shown in Table 3. The results showed that the FID of the samples generated by DCGANB was 38.9, indicating higher fidelity and diversity compared to those generated by traditional data augmentation methods.

In further testing and evaluating colony data, the generation structure of bacterial images is influenced by multiple factors, including the number of epochs (training times), the amount of DCGANB raw data, and the evaluation of FID parameter values. This study proposed and validated an interpretable quantitative framework for determining the optimal combination of the number of real samples (X), the number of synthetically generated samples (Y), and the number of training epochs (Z) for DCGAN under data-scarcity conditions, with the objective of minimizing the Fréchet Inception Distance (FID) and thereby improving the quality of the generated data. We logarithmized FID and constructed a second-order regression-based evaluation model that includes ln (X) and ln (Y) and performed interpolation fitting. Unlike pure empirical parameter adjustment calculation testing, pure empirical parameter adjustment greatly reduces computational resources and time. This method provides a reproducible and statistically verifiable decision-making basis. Using a stable parameterization setting, the target variable is logarithmized as follows:

(7) *fid =* ln(FID)

A nonlinear regression model is established, including parameters such as the actual dataset ln(X), the synthetic dataset ln(Y), and the number of cycles Z. Considering that when there are too few synthetic samples, the trained model may overfit the real data, resulting in a higher FID. When there too many synthetic samples, the distribution learned by the DCGAN model may deviate from the real distribution, leading to an increase in FID as well. Since this type conforms to a quadratic function structure, the model was established as a quadratic regression model.

(8) *fid* = *f*(*ln*(*Y*))

Under the conditions of determining ln(X) and Z parameters, the optimal parameters for the number of artificially synthesized samples Y are determined.

(9) *fid* = *β*_0_ +*β*_1_*ln*(*X*) + *β*_2_*ln*(*Y*) + *β*_3_ (*ln*(*Y*))^2^ + *β*_4_ *Z*

Let ln(Y) be y, and perform the maximum/minimum calculation on y.

(10) $$y=-\frac{\beta_{2}}{2\beta_{3}}$$

The optimal sample size Y* is calculated.

(11) Y* = *e^y^*

This time, we preferred to use factorial design for calculation, performing multiple combinations of repeated calculations. The calculation was performed with an Epoch of 700, and the results of the FID evaluation data are shown in Table 4. *β*_2_ was calculated as −11.086, *β*_3_ as 0.771, and y was further calculated as 7.186.

(12) Y* = *e^y^* = 1320.91

**Figure 10:**
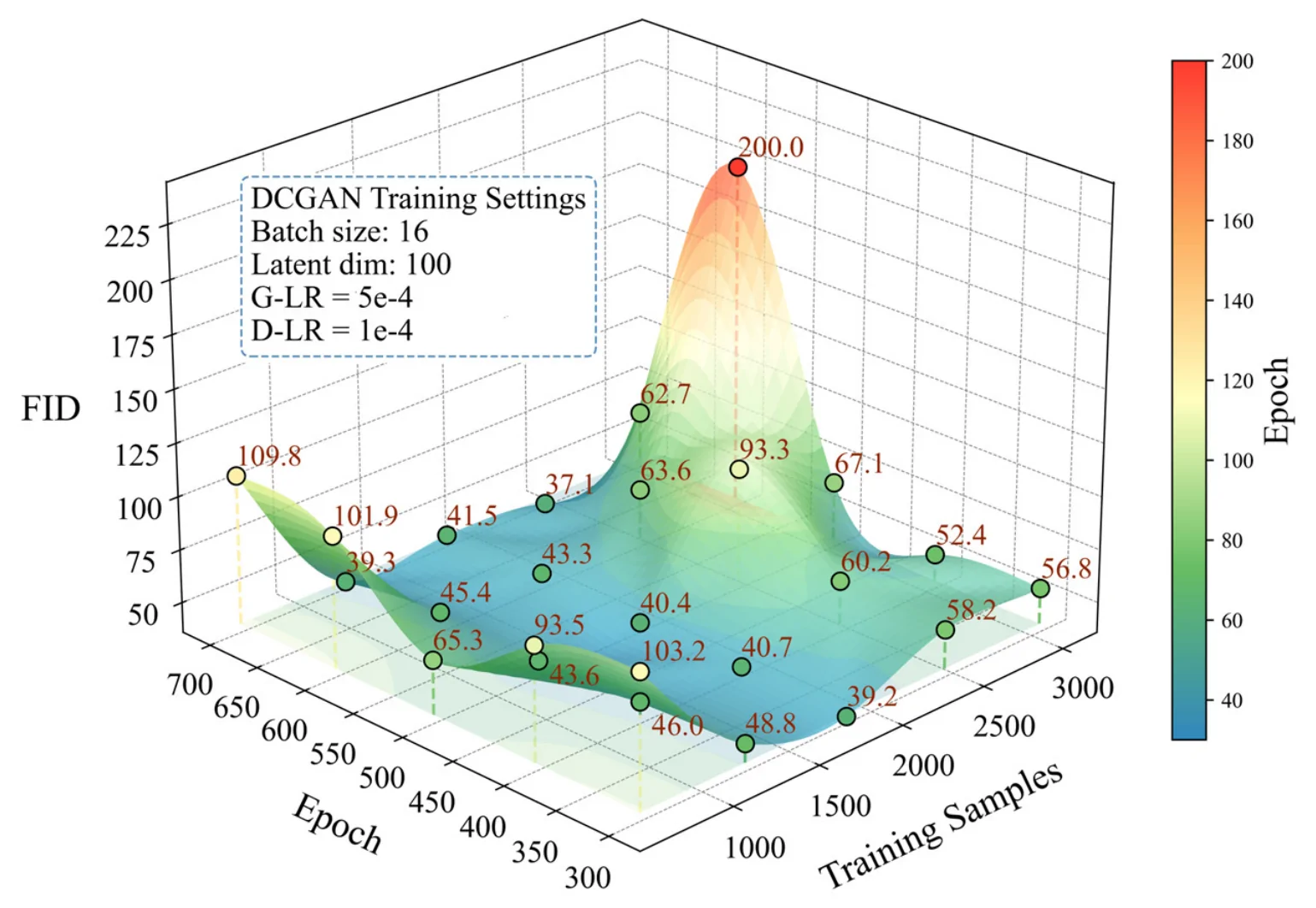
3D surface fitting of the binary regression model for DCGAN-B performance. FID scores are mapped against epochs and sample sizes under fixed hyperparameters, with color gradients highlighting model convergence and quality.

Therefore, based on the quadratic regression model, it was determined that the optimal effect is achieved when calculating the FID value with the addition of 1320 artificial samples. Meanwhile, we compared the data calculation effects of different Epochs, as shown in Figure 10. The study found that the FID value is the smallest in the range of 1100–1400, and the minimum value can reach 37.1, achieving the best optimization effect.

### 4.2. Experimental Verification and Analysis

**Figure 11:**
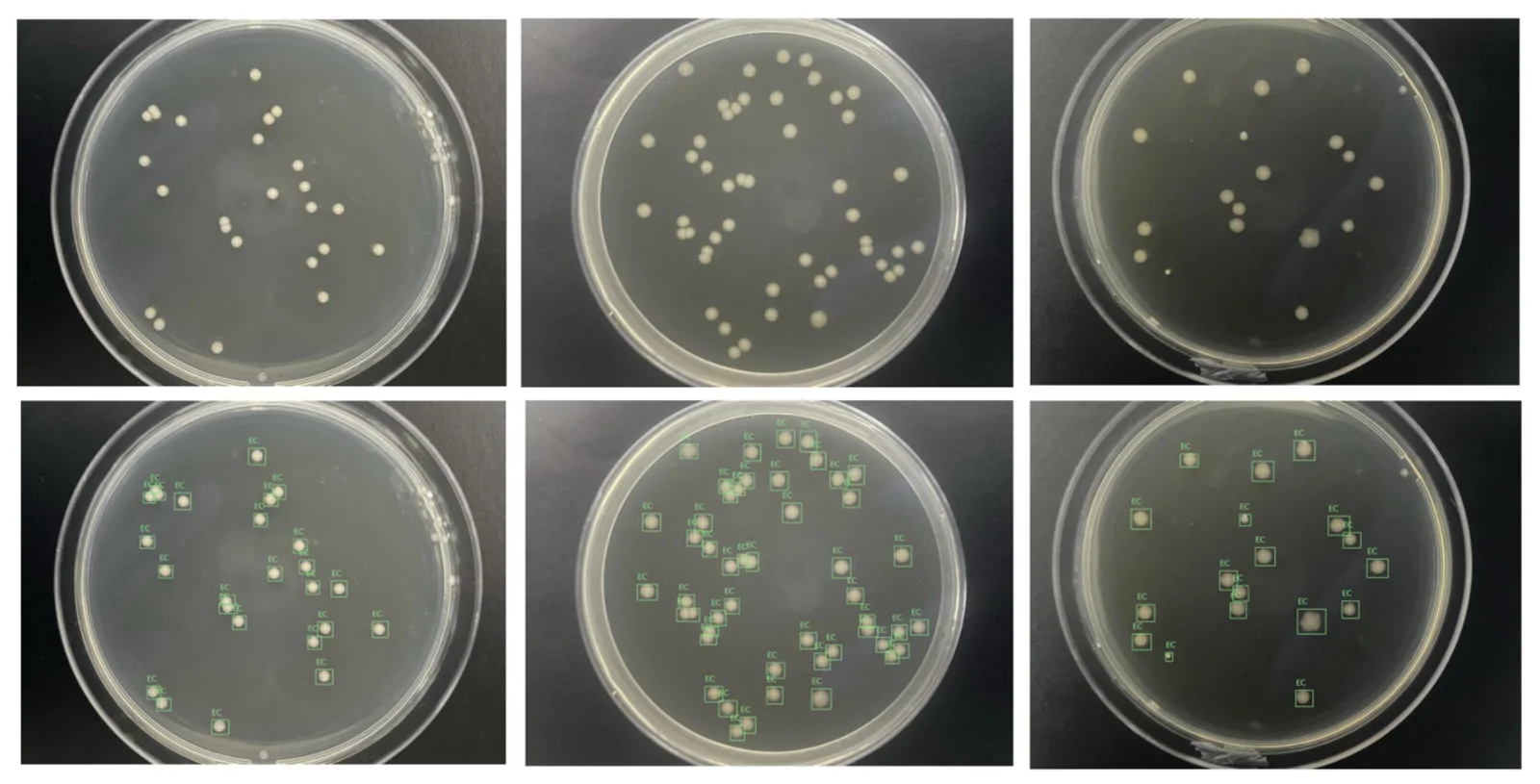
Example of microbial colony labeling on a self-cultured real dataset.

After evaluating the experimental results, we established an evaluation model and analyzed the optimal sample size. Further, we needed to experimentally verify the effect of increasing the sample size on image enhancement and recognition accuracy. We generated a D-DCGAN model to create an enhanced dataset. This dataset was used for training recognition. Two main testing methods were employed. We trained Faster R-CNN and YOLOv12 on three synthetic datasets for recognition data testing and compared the performance using different trained test datasets. We trained on the original dataset separately and on a combined dataset generated from the original dataset, following the optimal dataset size for binary models. We calculated the accuracy of test data recognition, which represents the percentage of bacterial colony recognition, to further evaluate the efficiency of data recognition. The labeled dataset of real plates is shown in Figure 11, including partial cutting data and the complete dataset of Petri dishes.

In this calculation, firstly, the original dataset of 236 was used for training and calculation, and all images were standardized. An edge enhancement algorithm based on median filtering was employed for preliminary image processing. Secondly, images from the original 236 samples, along with 600 and 900 DCGANB generated samples, were processed. Finally, images from 1320 DCGANB generated samples, which were obtained through second-order fitting to the optimal solution, were processed for testing of the analysis results. The mean average precision (mAP) and count results generated from the test dataset are shown in Table 5.

## 5. Discussion

This study proposed a microbial colony image enhancement method based on a composite generative adversarial network, termed D-DCGAN (Composite Adversarial Generative Network). By employing a three-stage data generation strategy and a secondary regression-based evaluation model, the proposed method can effectively expand microbial colony image datasets under small-sample conditions and substantially improve the recognition performance of downstream object detection models. The experimental results demonstrate that the synthetic images generated by D-DCGAN can effectively preserve the structural characteristics of real microbial colony images. Rather than simply applying conventional augmentation operations, such as rotation, cropping, or color transformation, the proposed approach learns the underlying data distribution of real microbial colony images through a generative model, thereby producing synthetic samples with diverse colony numbers, morphological characteristics, and spatial arrangements.

In recent years, with the rapid development of Generative Adversarial Network (GAN) theory and advances in computational hardware, GAN-based methods have been widely applied to medical image synthesis, data augmentation, and computer vision tasks. Previous studies have employed GANs to generate brain PET images with realistic structural characteristics [38], synthesize X-ray medical images [39], and generate retinal fundus images [40], demonstrating the potential of generative models for expanding training datasets under limited-data conditions. However, conventional GANs remain susceptible to several challenges during training, including mode collapse, training instability, and convergence difficulties. Their performance is also highly dependent on network architecture, loss functions, and parameter optimization strategies [41]. Consequently, recent studies have focused on improving GANs from various perspectives, including network architecture, training strategies, and supervision mechanisms, with the aim of achieving more stable training and higher-quality image generation [42,43].

Previous studies have demonstrated that improvements to different types of generative models can enhance the quality of synthesized images to some extent. For example, Nathanaël Carraz Rakotonirina et al. employed an Enhanced Super-Resolution Generative Adversarial Network (ESRGAN) for single-image super-resolution and improved the visual quality of generated images by incorporating a more densely connected residual feature extraction architecture [44]. However, such approaches primarily focus on image resolution enhancement and the recovery of local visual details, while their ability to model variations in colony number, spatial distribution, and inter-colony composition within microbial culture-plate images remains limited. Moreover, the generalization capability of generative models may still be affected by noise perturbations and variations in data distributions. Man Zhang et al. proposed a semi-supervised Blockwise architecture search method, which improves model efficiency by reducing the dependence on labeled data and optimizing network architecture [45]. This study demonstrates that reducing the dependence on annotated data is an important direction for the further application of generative models. Nevertheless, it mainly focuses on network architecture optimization and supervision efficiency, without specifically addressing the unique requirements of microbial culture-plate images, particularly local colony generation and global spatial arrangement. Yuwan Du et al. combined a generative adversarial network with a multiclass support vector machine for the classification and detection of foodborne pathogens under limited-sample conditions and achieved promising classification performance [46]. However, this approach primarily utilizes GANs to augment the data required for classification and subsequently employs SVM-based optimization for pathogen classification. Its data-generation strategy remains limited in modeling the spatial structure of complex colony images and the distributional characteristics of multiple colonies within a culture plate.

Compared with the aforementioned studies, the present study specifically addresses the structural characteristics of microbial culture-plate images by developing a hierarchical data-generation framework consisting of DCGAN-A and DCGAN-B. DCGAN-A first uses individual microbial colonies as the basic generation units and learns their local morphological and textural characteristics. Subsequently, a random spatial composition strategy is employed to arrange multiple generated colonies according to different spatial configurations, thereby producing intermediate images with varying colony numbers and spatial distributions. Based on these intermediate images, DCGAN-B further learns the global colony distribution characteristics of complete culture-plate images and performs additional image generation and refinement. This process establishes a hierarchical data augmentation mechanism that progresses from local colony feature modeling to global spatial distribution modeling. Consequently, the proposed generative framework is capable of learning not only the visual characteristics of individual colonies but also the relative spatial relationships among multiple colonies. In contrast to existing GAN-based approaches that primarily focus on image resolution enhancement, classification performance, or network architecture search efficiency, D-DCGAN is specifically designed to establish a structured and hierarchical data-generation process for microbial culture-plate images. Its primary advantage therefore lies in the explicit modeling of microbial colony features at both the local and global levels.

Quantitative experimental results further validate the effectiveness of the proposed method. The FID value decreased from 55.07 for randomly generated data to 38.9, while the SSIM increased to 0.6412 and the PSNR reached 26.7 dB. These results indicate that D-DCGAN can effectively learn the feature distribution of real microbial colony images while preserving a relatively high degree of structural similarity. Furthermore, the introduction of synthetic data significantly improved the performance of downstream object detection models, with both Faster R-CNN and YOLOv12 exhibiting enhanced detection performance. In particular, the recognition precision of Faster R-CNN increased from 0.872 to 0.975, accompanied by a substantial reduction in the mean absolute error (MAE). These findings demonstrate that high-quality synthetic data can not only alleviate the data scarcity commonly encountered in microbial imaging but also effectively improve the training performance of downstream recognition tasks.

Notably, our results indicate that increasing the amount of augmented data does not necessarily lead to continuous performance improvement. Excessive synthetic data may instead dilute or obscure discriminative features that are important for model learning. To quantitatively determine an appropriate amount of synthetic data, we established a secondary regression-based evaluation model using FID as the evaluation criterion and identified approximately 1320 synthetic images as the optimal augmentation level. This finding suggests that an optimal balance exists between real and synthetic data in limited-sample learning scenarios. An insufficient amount of synthetic data may fail to adequately expand the feature space, whereas excessive synthetic data may cause the model to learn features that deviate from the underlying real-data distribution, ultimately compromising both image-generation quality and downstream model performance.

Despite the promising experimental results, several limitations remain. First, the experimental dataset was derived exclusively from a single strain of *Escherichia coli*, whose colony morphology is relatively regular. Therefore, the generalization capability of the proposed model to multiple microbial strains, complex overlapping colonies, and varying culture conditions has not yet been systematically validated. Second, although the current random spatial composition strategy can simulate the spatial distribution of colonies within a culture plate, it does not fully account for biological phenomena associated with colony growth, such as mutual inhibition, boundary merging, and spatial competition. Consequently, certain irregularities may still occur in the generated images, particularly in regions near the boundaries of the culture plate. Third, the present study primarily relied on image-based metrics, including FID, SSIM, and PSNR, to evaluate the quality of the generated images. Although these metrics provide quantitative measures of visual similarity and image quality, they do not fully capture biological realism from a microbiological perspective. Therefore, further development of biologically informed evaluation criteria is warranted.

Future research will focus on constructing high-resolution datasets containing multiple microbial species and strains to systematically evaluate the applicability and generalization capability of D-DCGAN in more complex microbial imaging scenarios. Biological constraints, such as microbial growth kinetics and spatial growth patterns, could be incorporated into the generative framework, potentially through the integration of growth-dynamics models or diffusion-based generative models, to improve the biological plausibility of the synthesized images. Furthermore, the integration of synthetic data with downstream tasks, including instance segmentation and three-dimensional reconstruction, will be investigated to further assess the practical utility of the proposed approach in automated microbial detection and analysis systems. These efforts may provide more reliable and diverse data resources for intelligent microbial culture, automated colony analysis, and computer-assisted clinical diagnostic applications.

## 6. Conclusions

This article proposed an image enhancement model based on D-DCGAN, which was applied to the colony images of *Escherichia coli* culture dishes. This model is a composite DCGAN adversarial neural network algorithm that performs high-quality extensions on a limited colony dataset. We proposed a hybrid data generation method. In the first stage, DCGANA was used to generate images of single and composite colonies, generating features of various forms of small colonies. In the second stage, the culture dish image was randomly matched according to Gaussian random distribution. In the third stage, DCGAN-B was used to further synthesize and refine the composite culture-plate images, generating samples that preserve the characteristic distribution of the input images. Furthermore, to investigate the effects of multiple parameters on data-generation performance, a secondary evaluation model was developed to quantitatively determine the optimal number of synthetic images. Based on the evaluation results, the optimal augmentation level was identified as approximately 1320 synthetic colony images. The optimally trained DCGAN-B model was then used to generate the corresponding synthetic dataset, and its effectiveness was further validated through downstream detection experiments using Faster R-CNN and YOLOv12. Image enhancement effectively improved the precision of colony recognition to 0.975, which to some extent solved the problem of dataset construction in limited microbial culture dish colony data. In the future, based on this method, research can be conducted on high-resolution and diverse synthetic image enhancement directions.

## Figures and Tables

**Table 1:** DCGANA Training Structure Data Model Parameters.

Parameter	Numerical Value
Learning rate	Discriminator 0.0001, Generator 0.0002
Mini-batch	32
Normalization	Auto
Leaky ReLU	0.2
Adam optimizer	gradDecay = 0.6, sqGradDecay = 0.999
Regularization	weight_decay = 0.0001

**Table 2:** Training Parameters of DCGANB Data Model.

Parameter	Numerical Value
Learning rate	Discriminator 0.0001, Generator 0.0005
Mini-batch	16
Normalization	Auto
Leaky ReLU	0.1
Adam optimizer	gradDecay = 0.5, sqGradDecay = 0.999
Regularization	weight_decay = 0.0001

**Table 3:** Results of the Generated Dataset for Quantitative Evaluation.

Control Group	FID	SSIM	PSNR (dB)
Original data	0.00	1	/
Random data	55.07	0.4654	14.13
DCGANB data	38.9	0.6412	26.7

**Table 4:** Evaluation Results of FID Dataset.

Original Dataset (236)	Synthetic Dataset				
	400	1000	1600	2200	2800
FID	109.82	39.30	41.55	38.08	62.71

**Table 5:** Training Precision and MAE of Faster R-CNN and YOLOv12.

Dataset		Faster R-CNN		YOLOv12	
Raw dataset (236)	Synthetic dataset (0)	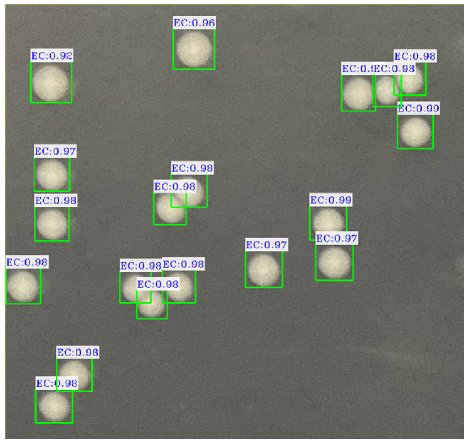	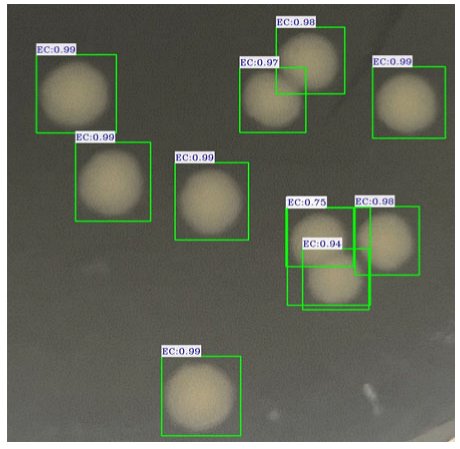	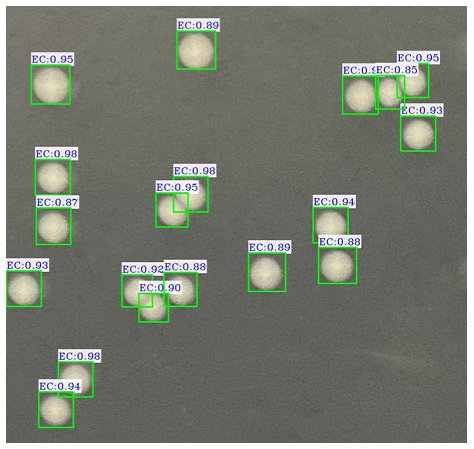	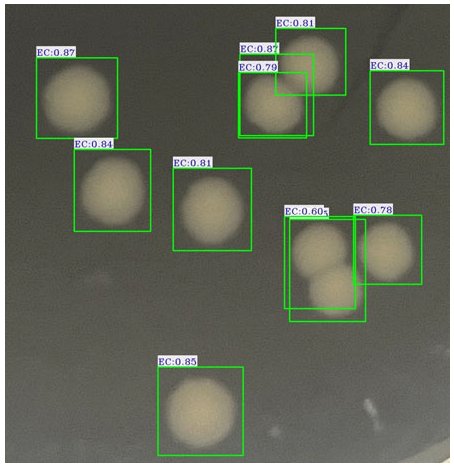
Raw dataset (236)	Synthetic dataset (600)	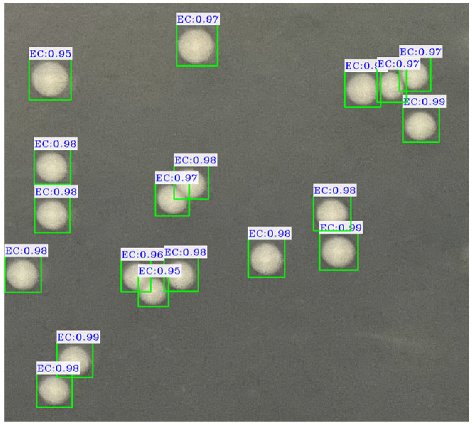	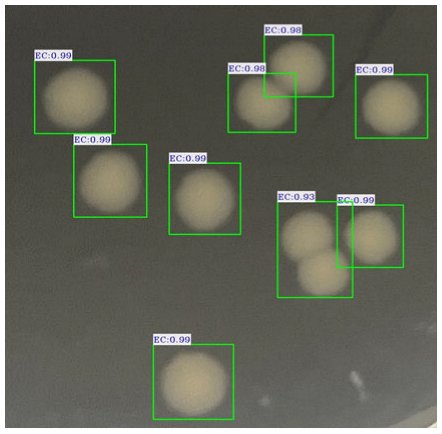	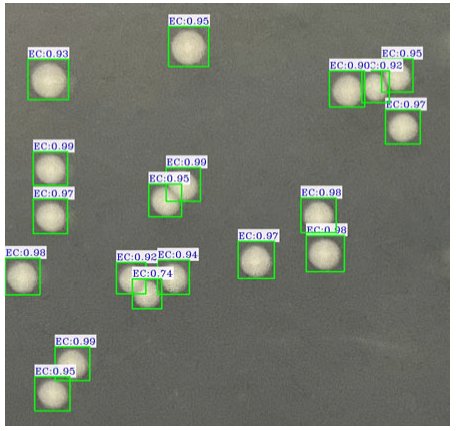	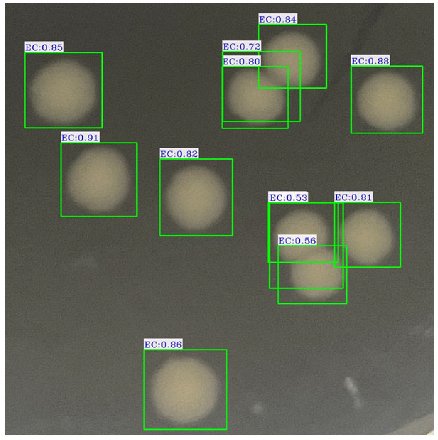
Raw dataset (236)	Synthetic dataset (900)	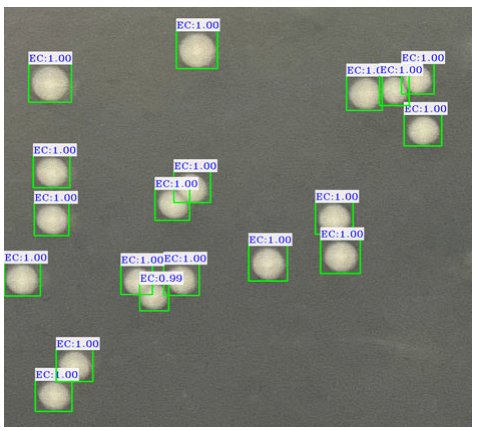	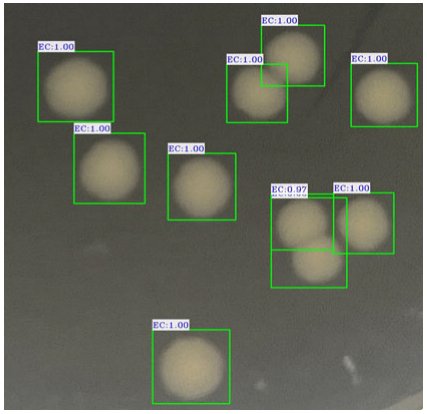	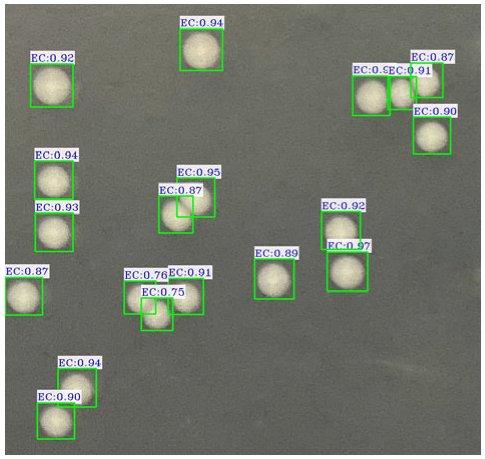	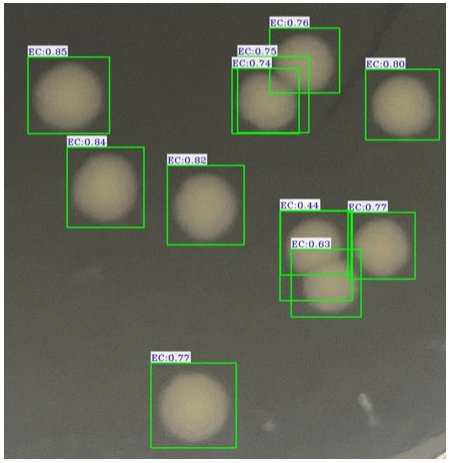
Raw dataset (236)	Synthetic dataset (1320)	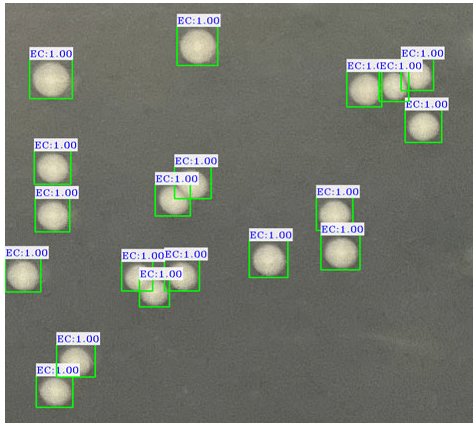	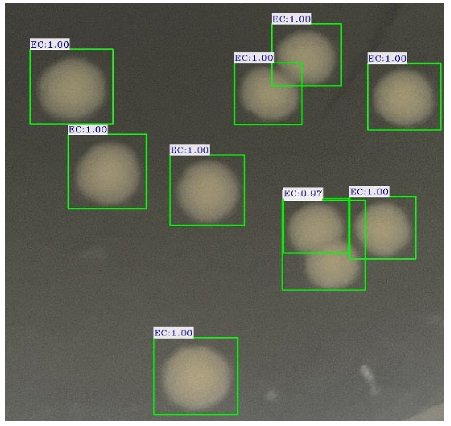	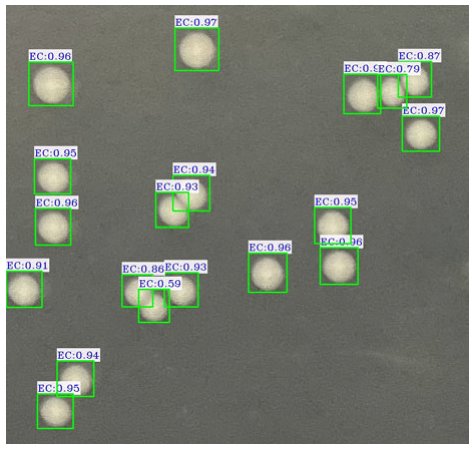	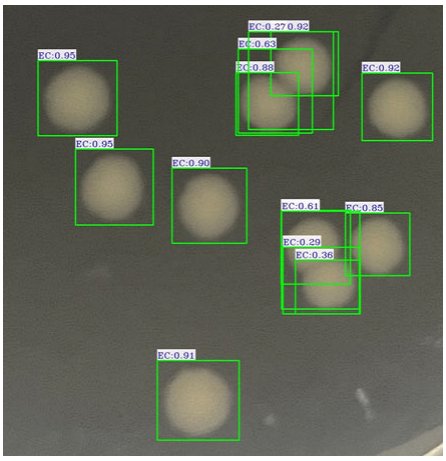

## Data Availability

The original contributions presented in this study are included in the article. Further inquiries can be directed to the corresponding author.
